# Leukotriene B_4_ is essential for lung host defence and alpha-defensin-1 production during *Achromobacter xylosoxidans* infection

**DOI:** 10.1038/s41598-017-17993-9

**Published:** 2017-12-15

**Authors:** Morgana K. B. Prado, Gisele A. Locachevic, Karina F. Zoccal, Francisco W. G. Paula-Silva, Caroline Fontanari, Joseane C. Ferreira, Priscilla A. T. Pereira, Luiz G. Gardinassi, Simone G. Ramos, Carlos A. Sorgi, Ana Lúcia C. Darini, Lúcia H. Faccioli

**Affiliations:** 1 Departamento de Análises Clínicas, Toxicológicas e Bromatológicas, Faculdade de Ciências Farmacêuticas de Ribeirão Preto, Universidade de São Paulo, Ribeirão Preto, São Paulo Brazil; 2 Departamento de Patologia e Medicina Legal, Faculdade de Medicina de Ribeirão Preto, Universidade de São Paulo, Ribeirão Preto, SP Brazil

## Abstract

Leukotriene B_4_ (LTB_4_) is essential for host immune defence. It increases neutrophil recruitment, phagocytosis and pathogen clearance, and decreases oedema and inflammasome activation. The host response and the role of LTB_4_ during *Achromobacter xylosoxidans* infection remain unexplored. Wild-type (129*sv*) and LTB_4_ deficient (*Alox5* ^−/−^) mice were intratracheally infected with *A. xylosoxidans*. Wild-type 129*sv* infected mice survived beyond the 8^th^ day post-infection, exhibited increased levels of LTB_4_ in the lung on the 1^st^ day, while levels of PGE_2_ increased on the 7^th^ day post-infection. Infected *Alox5* ^−/−^ mice showed impaired bacterial clearance, increased lung inflammation, and succumbed to the infection by the 7^th^ day. We found that exogenous LTB_4_ does not affect the phagocytosis of *A. xylosoxidans* by alveolar macrophages *in vitro*. However, treatment of infected animals with LTB_4_ protected from mortality, by reducing the bacterial load and inflammation via BLT_1_ signalling, the high affinity receptor for LTB_4_. Of importance, we uncovered that LTB_4_ induces gene and protein expression of α-defensin-1 during the infection. This molecule is essential for bacterial clearance and exhibits potent antimicrobial activity by disrupting *A. xylosoxidans* cell wall. Taken together, our data demonstrate a major role for LTB_4_ on the control of *A. xylosoxidans* infection.

## Introduction

Cystic fibrosis (CF) is a genetic disease caused by mutations in the cystic fibrosis transmembrane conductance regulator (CFTR), an ion-channel responsible for the transport of ions across the epithelial barrier^1^. The major CF complication is the persistent colonisation of the respiratory tract by opportunistic microorganisms, such as *Achromobacter xylosoxidans* ^2,3^. The bacillus produces virulence factors, exhibits clearance-evading mechanisms, and may resist to multiple classes of antibiotics^4^. It has been associated with the exacerbation of pulmonary symptoms and rapid decline in lung function^5^. CF patients have increased levels of granulocyte colony stimulating factor (G-CSF), interleukin (IL)-6, tumour necrosis factor (TNF)-α; and eicosanoids (EICs) such as leukotriene B_4_ (LTB_4_) and prostaglandin E_2_ (PGE_2_), compared to healthy controls^6–8^. Patients with chronic *A. xylosoxidans* infection produce high amounts of TNF-α in the sputum^7^; furthermore, components of *A. xylosoxidans* cell wall induce pro-inflammatory cytokine production by lung cells, monocytes and macrophages *in vitro* ^9–11^.

EICs are lipid mediators derived from the metabolism of arachidonic acid (AA) and related polyunsaturated fatty acids (PUFAs), present in membrane phospholipids. Three major enzymatic pathways–driven by the lipoxygenases (LOs), cyclooxygenases (COXs) and cytochrome P450 (CYP) epoxyhydrolases—convert free AA to generate leukotrienes (LTs), prostaglandins (PGs), epoxyeicosatrienoic acids (EETs) and hydroxyeicosatetraenoic acids (HETEs)^12^. Downstream mediators of 5-LO protect mice from infections with *Klebsiella pneumoniae* ^13^, *Mycobacterium tuberculosis* ^14^, *Streptococcus pyogenes* ^15^. LTB_4_ induces leukocyte recruitment^16^, increases phagocytosis and pathogen killing^15,17^, and increases antimicrobial peptide (AMP) production, like α-defensin-1^18^. Recently, our group reported that LTB_4_ reduces lung inflammation and prevents scorpion envenomation-induced death by down-regulating inflammasome activation and IL-1β production^19^. Despite the clinical importance of *A. xylosoxidans*, whether LTB_4_ elicits protective mechanisms against this bacterial infection still needs to be elucidated.

Here, we investigated the impact of 5-LO deficiency on lung inflammation, bacterial clearance and mortality in *A. xylosoxidans*-induced pneumonia. We observed that *Alox5* ^−/−^ mice are more susceptible to *A. xylosoxidans* infection than the 129*sv* wild-type mice. They exhibited increased mortality, higher bacterial burden and augmented lung inflammation and oedema. Furthermore, we determined that LTB_4_ protected against *A. xylosoxidans* infection, as intranasal administration of LTB_4_ to *Alox5* ^−/−^ mice reduced bacterial burden and lung oedema, increased neutrophil recruitment and prevented death. To confirm that LTB_4_ signalling is essential for lung protection during *A. xylosoxidans* infection, we treated infected 129*sv* mice with LTB_4_ receptor 1 antagonist (BLT1) and observed increased bacterial burden and oedema in the lungs, as well as higher mice mortality. Importantly, we demonstrated that LTB_4_ was fundamental for α-defensin-1 expression in infected alveolar macrophages *in vitro*, and in the lungs of *A. xylosoxidans* infected mice. This peptide is crucial for *A. xylosoxidans* clearance, once silencing of α-defensin-1 mRNA abrogated killing of *A. xylosoxidans* induced by LTB_4_. Using transmission electron microscopy, we confirmed that α-defensin-1 reduced *A. xylosoxidans* viability by disrupting the bacterial cell wall.

## Results

### Infection with *A. xylosoxidans* induces the production of eicosanoids

To understand the host response to infections with *A. xylosoxidans*, we first established a murine experimental lung infection model with this pathogen. The moving average interpolation method^20^ and biometric tables for median-lethal dose (LD_50_) calculation^21^ were used to predict the lethal and sublethal LD_50_ inoculums of *A. xylosoxidans* in 129*sv* mice. The median lethal and sublethal doses were estimated as 6.2 × 10^8^ and 2 × 10^8^ bacilli, respectively (Supplementary Table 1).

**Figure 1:**
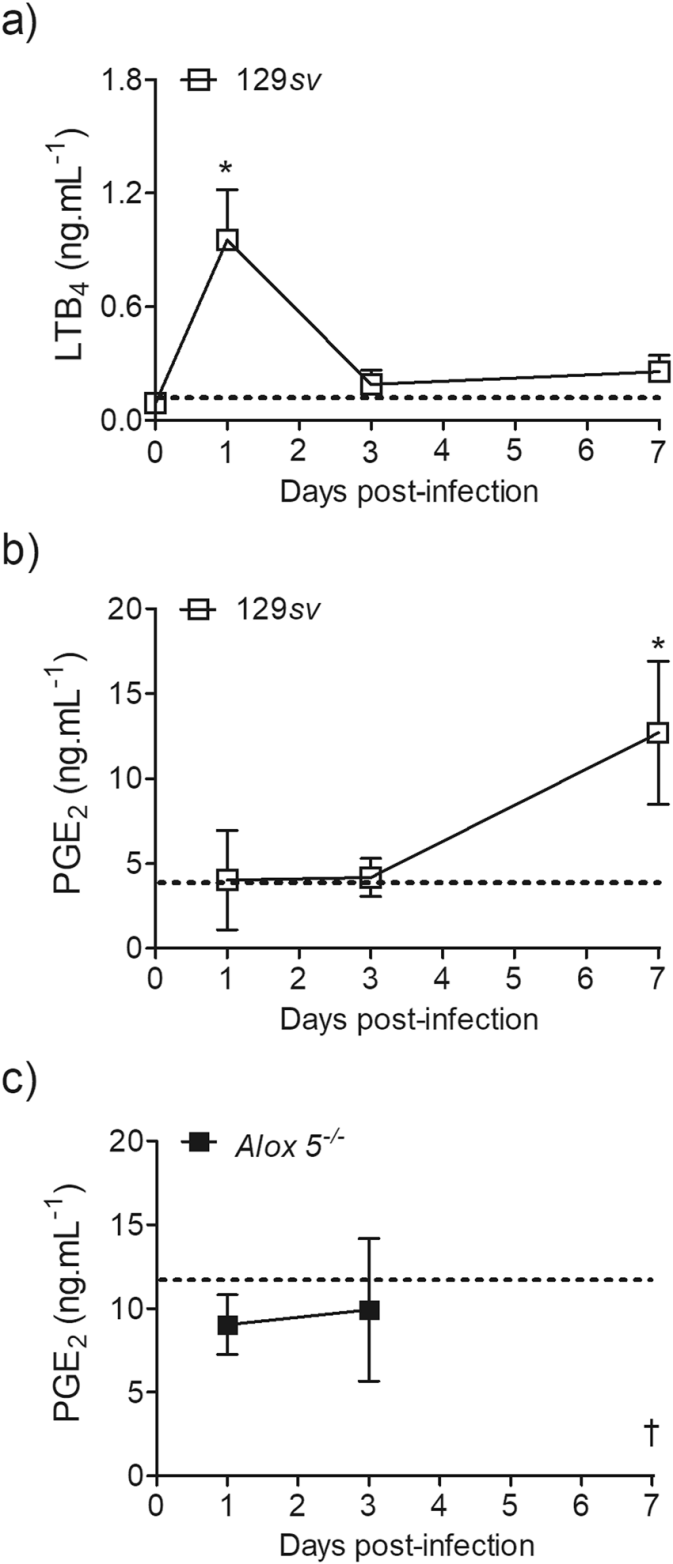
*A. xylosoxidans* infection induces LTB_4_ and PGE_2_ production in the lung. 129*sv* wild-type (**a**,**b**) or *Alox5* ^−/−^ (**c**) mice were inoculated i.t. with 100 μL of PBS (dashed line) or with sublethal inoculum of *A. xylosoxidans* (2 × 10^8^ in 100 μL of PBS). Lung tissue was collected at 1, 3 and 7 days post-infection (all *Alox5* ^−/−^ died within 7 days [†]) and processed for LTB_4_ and PGE_2_ measurements in the lung parenchyma. Data are mean ± SEM from one representative experiment (n = 4–5 mice group for each time-point). *p < 0.05 for uninfected (UN) *versus* infected mice at the same time-point by a one-way analysis of variance with a Newman-Keuls multiple comparison post-test.

Using a sublethal inoculum (2 × 10^8^ bacilli) of *A. xylosoxidans*, we first assessed the production of PGE_2_ and LTB_4_ induced by infection in the lung parenchyma. In the lungs of 129*sv* mice, LTB_4_ levels peaked on the 1^st^ day post-infection (Fig. 1a), and declined thereafter to reach basal levels by the 3^rd^ day. However, detectable levels of PGE_2_ were observed only on the 7^th^ day post-infection (Fig. 1b). Interestingly, *Alox5* ^−/−^ mice produced similar amounts of PGE_2_ between days 1 and 3 post-infection, which did not differ from the uninfected mice (Fig. 1c). However, as all *Alox5* ^−/−^ mice succumbed to the infection within 5 days, we could not measure PGE_2_ production after this time-point (Fig. 1c). Irrespective of the infection, *Alox5* ^−/−^ mice demonstrated higher production of PGE_2_ than the 129*sv* mice (Fig. 1b,c).

### 5-LO deficiency increases mortality, bacterial burden and lung inflammation

**Figure 2:**
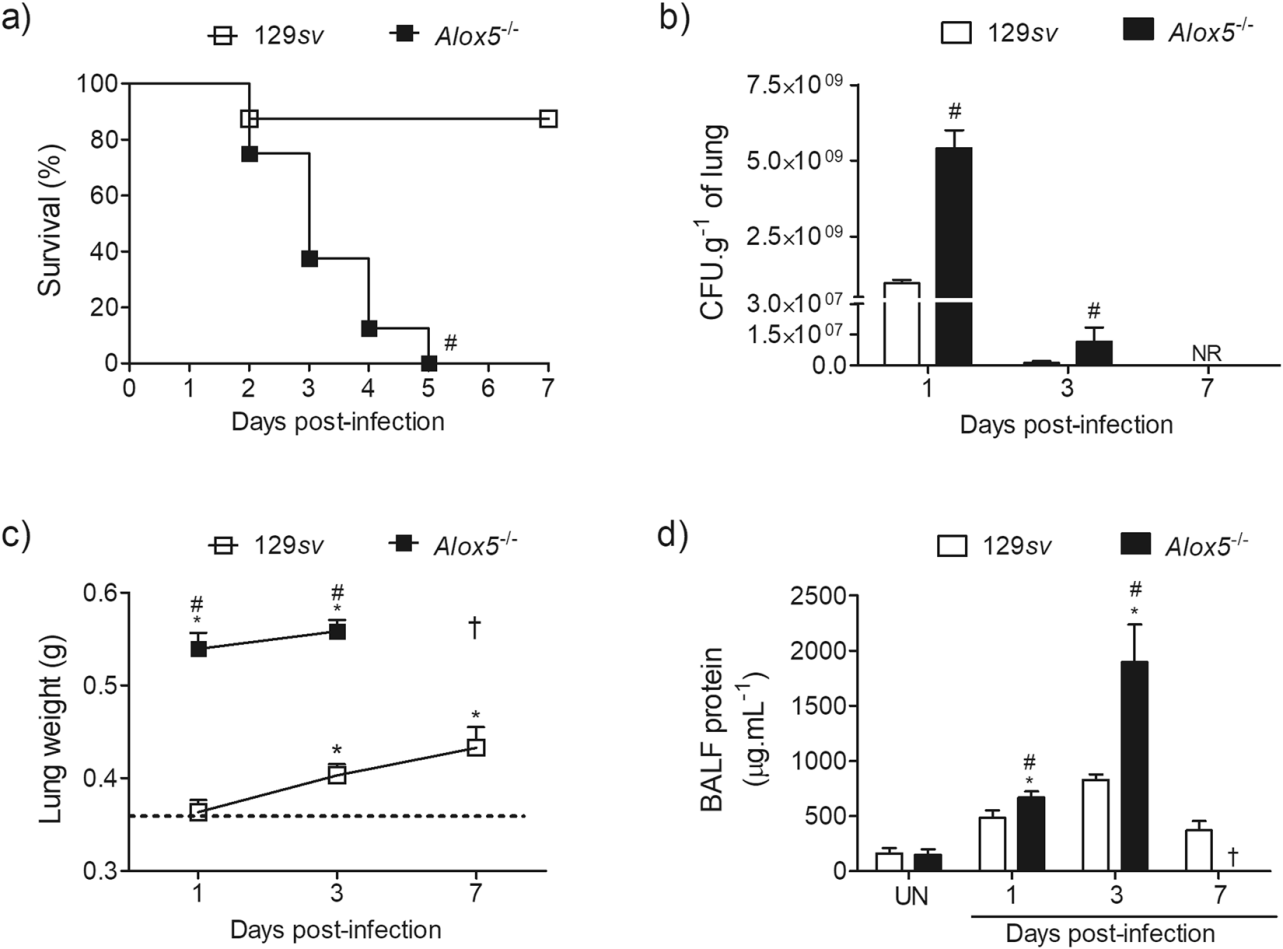
5-LO deficiency impairs bacterial clearance, and increases lung oedema and mortality. Wt and *Alox5* ^−/−^ mice were i.t. infected with sublethal inoculum of *A. xylosoxidans* (2 × 10^8^/100 μL in PBS) for survival determination until 7 days post-infection (n = 8 mice per strain) (**a**). Log-Rank test was used to compare survival between the strains. Comparison of bacterial burden (**b**), lung weight (**c**), and BALF protein content (**d**). Uninfected (UN) mice receiving i.t. 100 μL of PBS were used as negative control (dashed lines). Data are mean ± SEM of three independent experiments (n = 5–14 mice per group at each time-point). *p < 0.05 for UN *versus* infected animals at each time-point and for both strains, ^#^ *p* < 0.05 for wt *versus Alox5* ^−/−^ *m*ice using student’s *t*- test (**b**) or one-way analysis of variance with Newman-Keuls multiple post-test (**c**,**d**). ^†^Mice died, NR: not recovered (using this method). BALF: bronchoalveolar lavage fluid.

Previous investigations indicate that *Alox5* ^−/−^ mice are more susceptible to infections and envenomation^17,19^. To evaluate the role of 5-LO products in the infection with *A*. *xylosoxidans*, 129*sv* and *Alox5* ^−/−^ mice were infected with sublethal *A*. *xylosoxidans* inoculums. We observed that the infection was robust but self-limiting in the 129*sv* mice, as no CFUs were recovered 7 days post-infection (Fig. 2b). Downstream products of 5-LO actively affect the course of infection with *A*. *xylosoxidans*, revealed by the increased mortality of *Alox5* ^−/−^ mice (Fig. 2a). Following infection, 100% of the *Alox5* ^−/−^ mice died within the first 5 days, while 85% of the wild-type mice survived 7 days post-infection (Fig. 2a) (wild-type animals survived for at least 14 days post-infection, the last observed time-point, data not shown).

**Figure 3:**
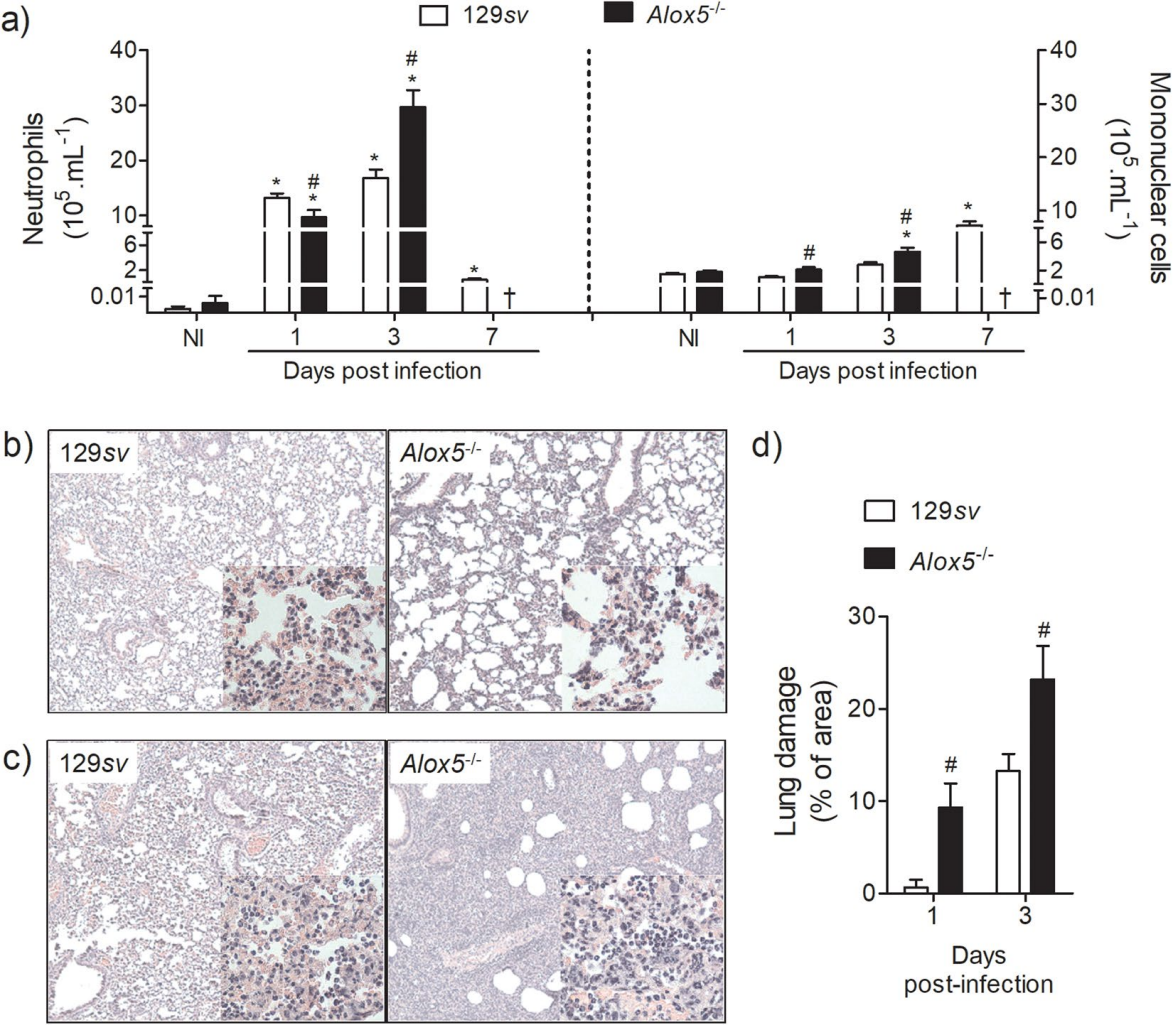
*Alox5* ^−/−^ mice exhibit pronounced lung inflammation following pulmonary infection with *A. xylosoxidans*. Neutrophil and mononuclear cell counts in the BALF of *A. xylosoxidans* (2 × 10^8^/100 μL of PBS) or PBS inoculated 129*sv* and *Alox5* ^−/−^mice at 1, 3 and 7 days post sublethal infection (**a**). Representative HE stained lung sections at day 1 (**b**) and 3 post-infection (**c**) (100× and 1000× [in inset] resolution, scale bar: 100 µm). Percentage of immune cell infiltrated area corresponding to the extent of lung damage (**d**). Data are mean ± SEM of two independent experiment (n = 5–10 mice per group at each time-point). Differences were considered significant with *p* < 0.05 calculated using one-way ANOVA (Newman-Keuls multiple comparison test) (**a**). *p < 0.05 for untreated (UN) *versus* infected, ^#^ *p* < 0.05 for wt *versus Alox5* ^−/−^ by student’s t-test (**d**) or one-way analysis of variance with Newman-Keuls multiple comparison (**a**), ^†^mice died.

To investigate the potential mechanisms resulting in early mortality, we determined bacterial burden and inflammation in the lungs of both mouse strains. The *Alox5* ^−/−^ mice exhibited a 5- and 9-fold higher lung bacterial burden compared to the wild-type, at days 1 and 3 post-infection, respectively (Fig. 2b). We did not find bacteria in the spleen and blood of both infected mouse strains (data not shown). Lung inflammation was more pronounced in the *Alox5* ^−/−^ mice, which exhibited an increase of 47% in lung weight compared to an increase of 38% in 129*sv* mice (Fig. 2c). BALF protein concentrations increased 129% in *Alox5* ^−/−^ mice, while 129*sv* mice exhibited an increase of 39% at days 1 and 3 post-infection (Fig. 2d). Compared to 129*sv*, the *Alox5* ^−/−^ mice showed a small reduction in the BALF neutrophil counts at day 1, but a significant increase in neutrophils at day 3 post-infection (Fig. 3a). The increased inflammation in the lungs of *Alox5* ^−/−^ mice was confirmed by histological analysis, showing a massive leukocyte infiltration in the lung parenchyma (Fig. 3b,c), and increased damage of the lung architecture (Fig. 3d).

### Absence of 5-LO enhances TNF-α, IL-1α and MIP-1α in infected lungs

**Figure 4:**
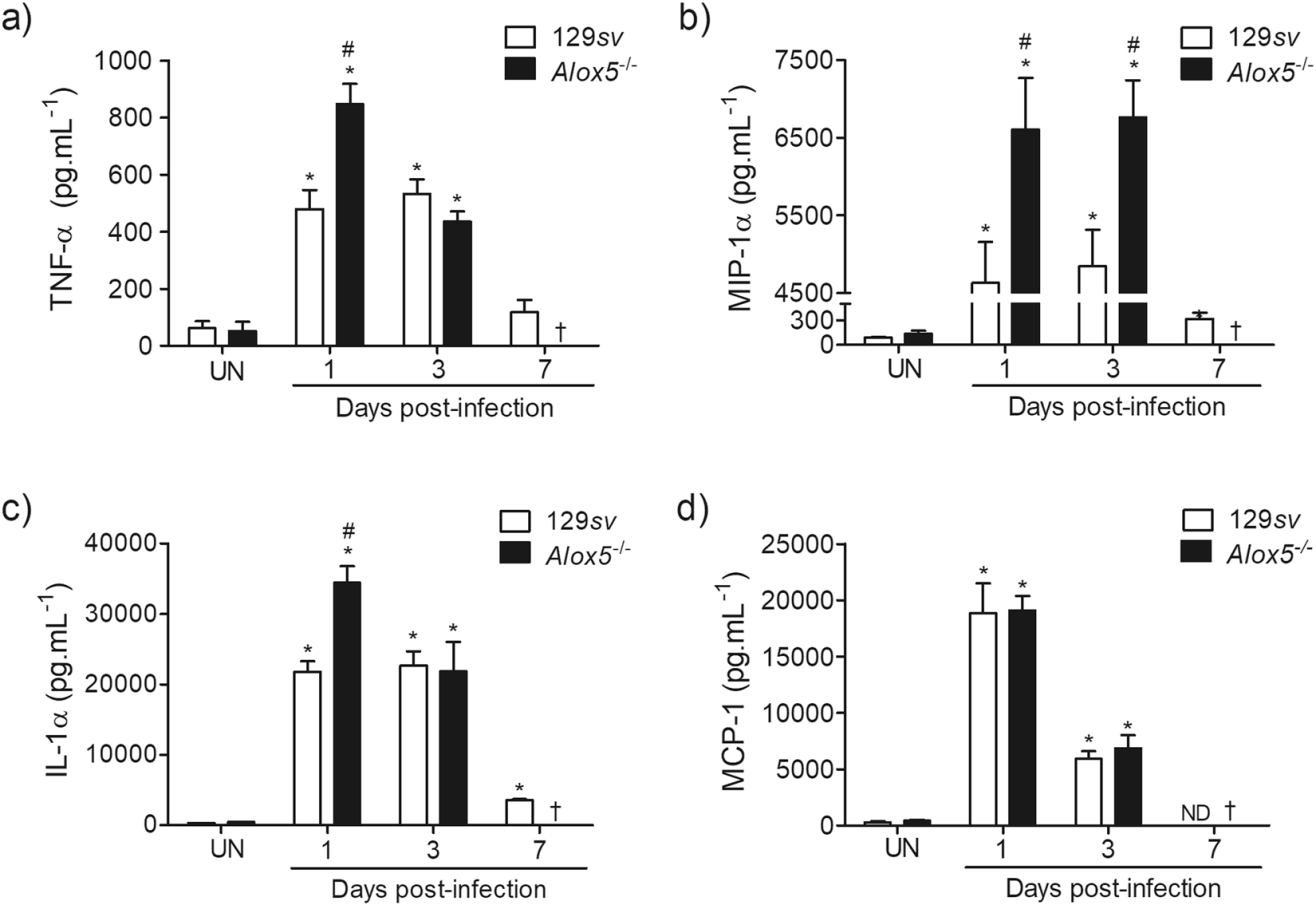
Impact of 5-LO deficiency on cytokine and chemokine production in the lungs of *A. xylosoxidans*-infected mice. TNF-α (**a**), MIP-1α (**b**), IL-1α (**c**), and MCP-1 (**d**) concentrations at 1, 3 and 7 days following infection. The data are mean ± SEM of three independent experiments (n = 5–10 mice per group at each time-point). *p < 0.05 for untreated (UN) *versus* infected mice, ^#^ *p* < 0.05 for wt *versus Alox5* ^−/−^ using one-way analysis of variance and Newman-Keuls multiple comparison test.

Inflammation is a crucial process in host response against aggressors, and is mediated by the action of pro-inflammatory cytokines, chemokines and eicosanoids, which promote leukocyte recruitment and oedema^19,22,23^. Therefore, we next assessed the levels of cytokine and chemokine in lung parenchyma of infected animals. At the 1^st^ and 3^rd^ day, infection with *A. xylosoxidans* induced significant release of TNF-α, MIP-1α, IL-1α and MCP-1 in both 129*sv* and *Alox5* ^−/−^ mice (Fig. 4). Compared to 129*sv* mice, the levels of TNF-α, IL-1α, and MIP-1α were 76%, 58%, and 42% higher in *Alox5* ^−/−^ mice at the 1^st^ day post-infection (Fig. 4a–c). However, at the 3^rd^ day post-infection, only levels of MIP-1α were higher in the *Alox5* ^−/−^ mice (Fig. 4b). The MCP-1 production was similar in both strains at all observed time-points (Fig. 4d). On the 7^th^ day post-infection, these cytokines were detected at very low levels in the lungs of 129*sv* mice.

### Intranasal LTB_4_ restores lung innate immune response and reduces mortality in the Alox5^−/−^ infected mice

**Figure 5:**
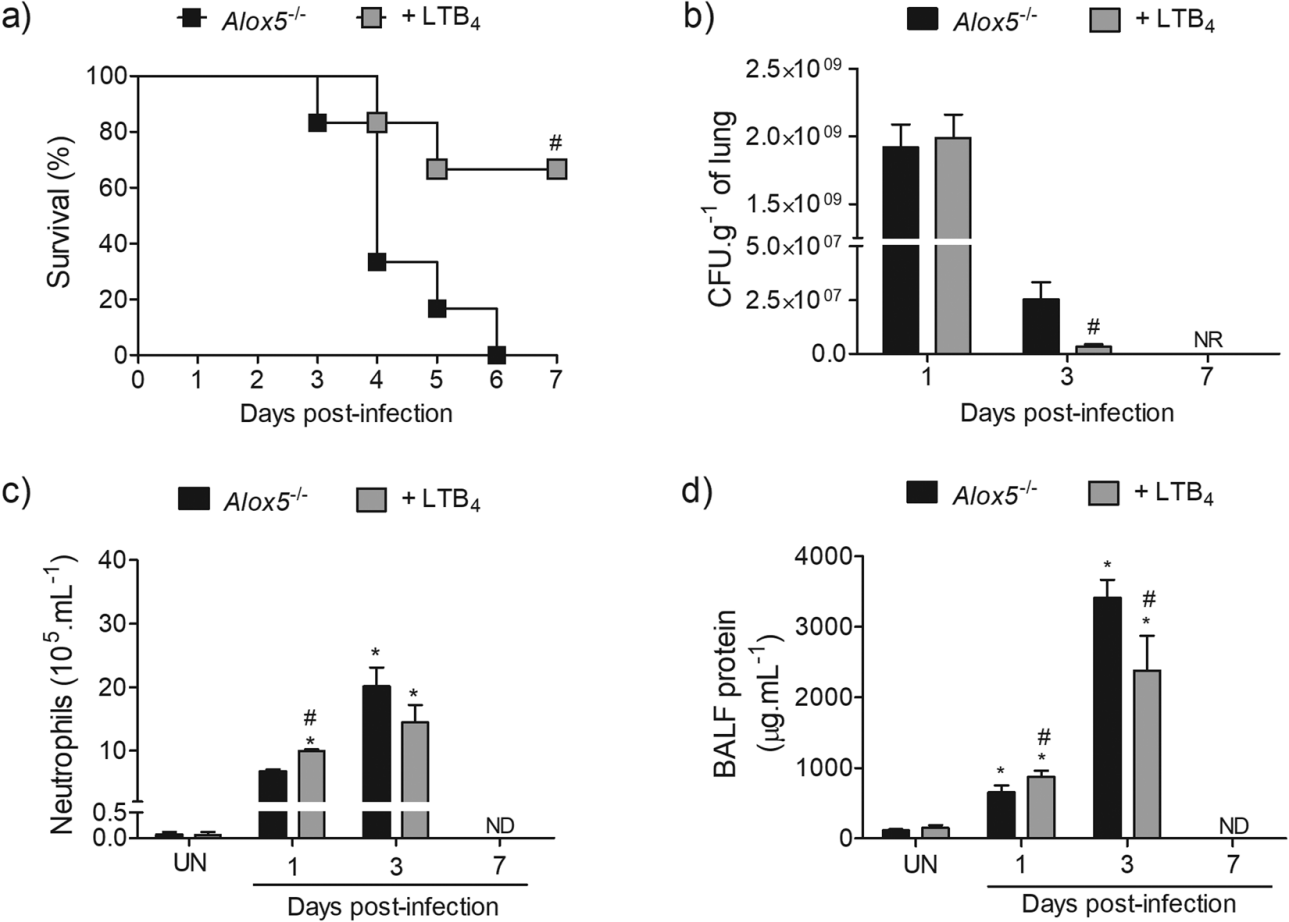
LTB_4_ treatment reduces susceptibility of *Alox5* ^−/−^ mice to pulmonary infection with *A. xylosoxidans*. Survival rate of infected *Alox5* ^−/−^ mice with or without LTB_4_ treatment was evaluated for 7 days (**a**) (n = 6 total), and *p* < 0.05 considered significant using Log-Rank test. Quantification of bacterial burden (**b**), BALF neutrophil counts (**c**) and protein content (**d**) at 1 and 3 days post-infection. The data are mean ± S.E.M of three independent experiment (n = 4–10 mice per group at each day). *p < 0.05 for uninfected (UN) *versus* infected mice, ^#^ *p* < 0.05 for *Alox5* ^−/−^ *versus Alox5* ^−/−^ + LTB_4_ using one-way analysis of variance (Newman-Keuls multiple comparison test). BALF: bronchoalveolar lavage fluid.

As the *Alox5* ^−/−^ mice were more susceptible to infection than the 129*sv*, we evaluated the effect of LTB_4_ treatment on the genetically modified mice. Infected *Alox5* ^−/−^ mice treated with LTB_4_ exhibited 66% survival at the 7^th^ day post-infection, compared to 0% survival of vehicle-treated infected *Alox5* ^−/−^ mice (Fig. 5a). At the 3^rd^ day post-infection, LTB_4_-treated mice showed significantly lower bacterial burdens (Fig. 5b), and lower BALF protein extravasation (Fig. 5d). As expected, LTB_4_ treatment increased neutrophil recruitment at the 1^st^ day post-infection (Fig. 5c), while no differences were observed in the mononuclear cell recruitment (data not shown).

### Blocking of BLT1 signalling impairs lung innate immune response and increases mortality in 129*sv* mice

**Figure 6:**
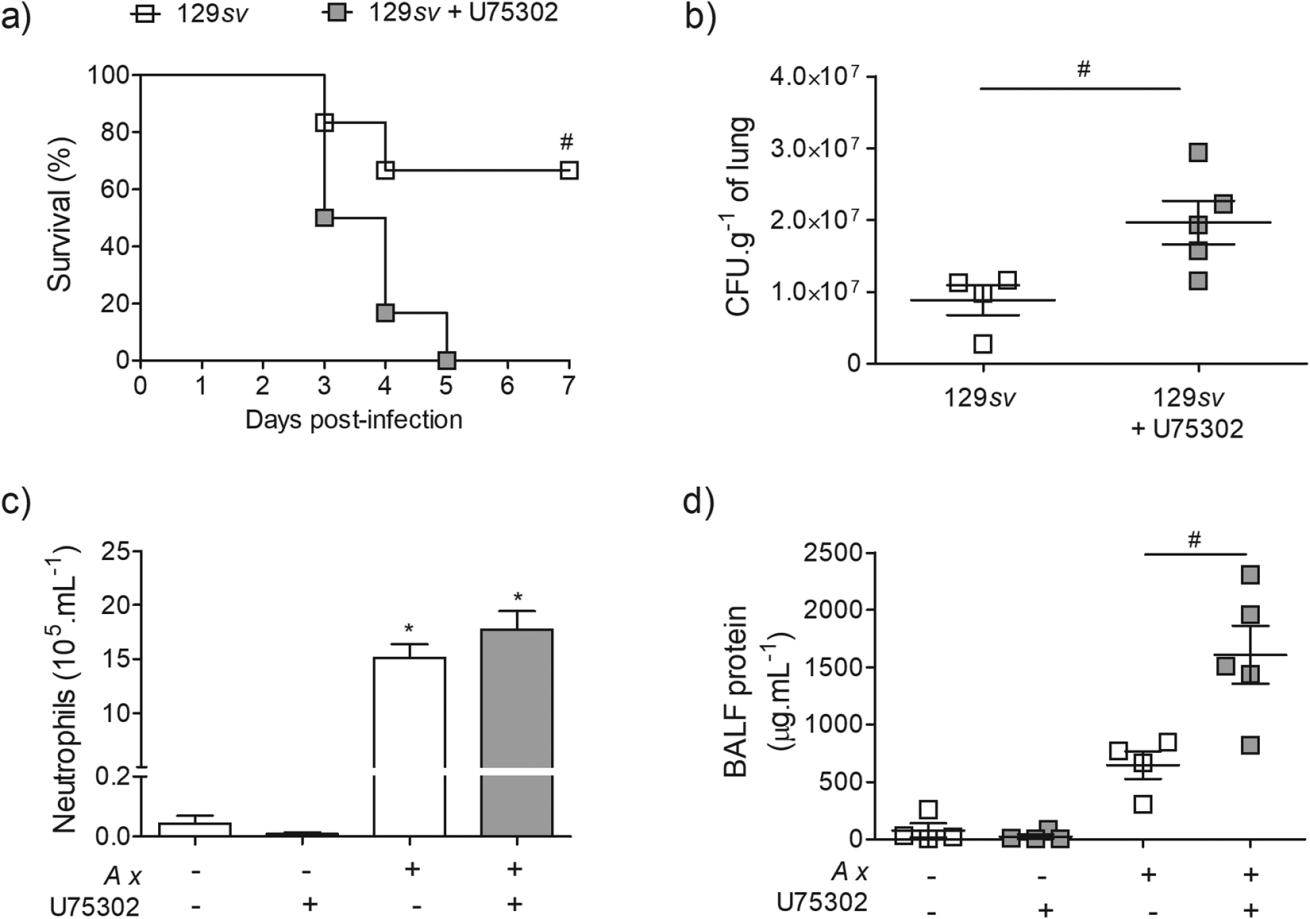
Blockade of BLT_1_ signalling increases susceptibility of 129*sv* mice to *A. xylosoxidans* infection. Survival rate for infected wt mice with or without BLT_1_ antagonist treatment was evaluated for 7 days (**a**) (n = 6 total), and *p* < 0.05 considered significant using Log-Rank test. Quantification of bacterial burden (**b**), BALF neutrophil counts (**c**) and protein content (**d**). Data are mean ± S.E.M of one representative experiment (n = 4–6 mice per group at each day). *p < 0.05 for untreated (UN) *versus* infected mice, ^#^ *p* < 0.05 for 129*sv versus* 129*sv* + BLT_1_ antagonist U-75302 using one-way ANOVA (Newman-Keuls multiple comparison test). BALF: bronchoalveolar lavage fluid.

Since LTB_4_ administration protected the *Alox5* ^−/−^ infected mice, we next investigated the effect of BLT_1_ signalling blockade on the 129*sv* mice. Our results demonstrate that the treatment of 129*sv*-infected mice with U-75302, a BLT_1_ antagonist, resulted in 100% mortality (Fig. 6a). At the 3^rd^ day post-infection, the treatment increased lung bacterial burden (Fig. 6b), without affecting neutrophil recruitment in BALF (Fig. 6c). Interestingly, BLT_1_ antagonist increased the protein concentration in the BALF of infected mice (Fig. 6d).

### LTB_4_ increases killing of *A. xylosoxidans* and *Defa1* expression in alveolar macrophages

**Figure 7:**
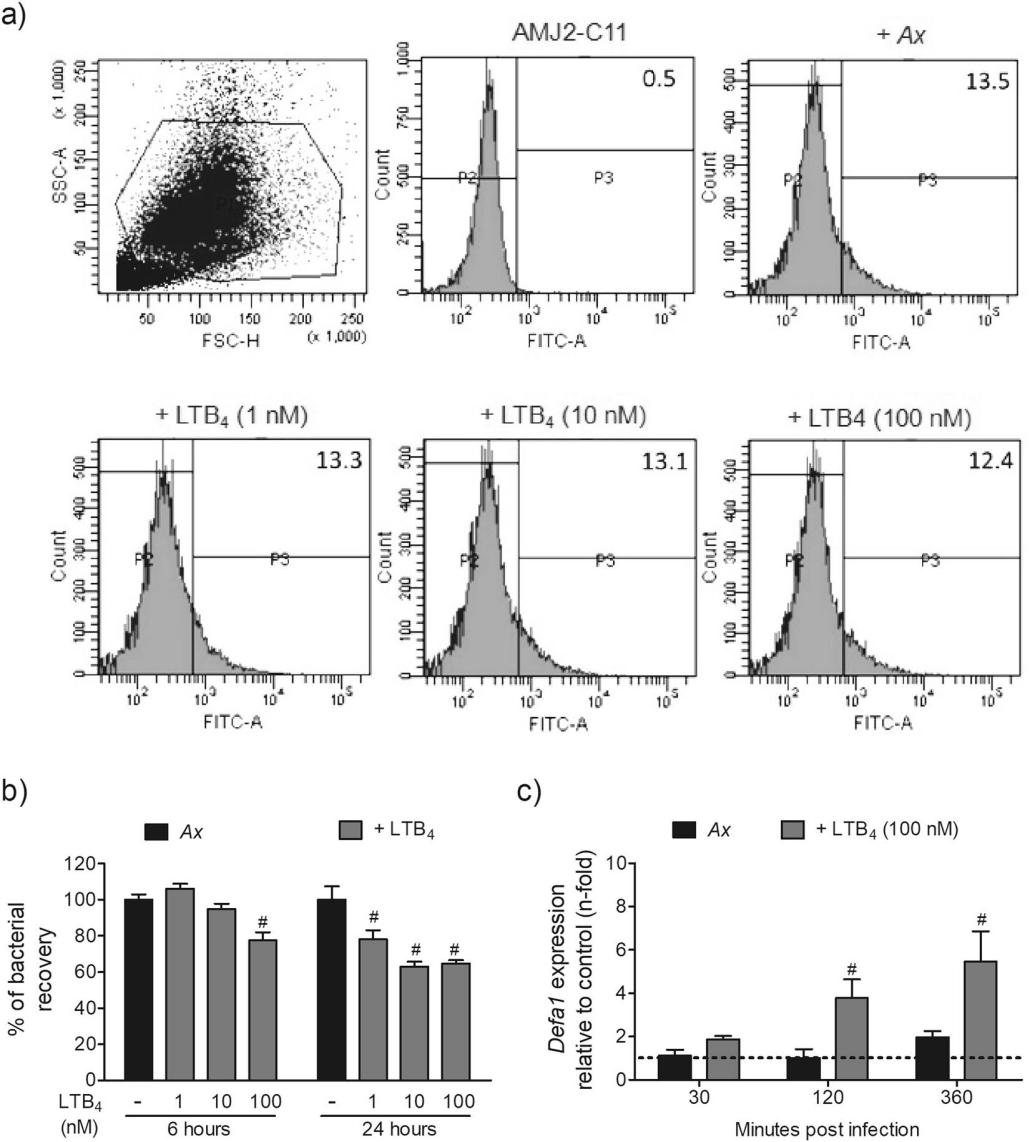
LTB_4_ increases killing of *A. xylosoxidans* and *Defa-1* expression in alveolar macrophages without affecting phagocytosis. AMJ2-C11 was treated or not with crescent concentrations of LTB_4_ (1, 10 and 100 nM). Cells were infected with FITC-labelled *A. xylosoxidans* (MOI 50:1) and incubated for 2 h at 37 °C, 5% CO_2_. After this period, the cells were washed, incubated with trypan blue 0.4% pH 4.4 and submitted to flow cytometry analysis for phagocytosis determination (**a**). Representative histogram out of four independent experiments is shown (n = 12). For bacterial killing assay, cells were infected with *A. xylosoxidans* (MOI 5:1) and incubated for 6 or 24 h at 37 °C, 5% CO_2_. Then, the cells were lysed with saponin (0.05%), mixed with 10 μL of a resazurin solution (0.5 mg/mL) and after 8 h at 37 °C the RFU was measured by fluorimeter microplate reader (**b**). One representative experiment out of three is shown as mean ± S.E.M. (n = 4–5 wells), ^#^ *p* < 0.05 for untreated *versus* LTB_4_ treated cells, using one-way analysis of variance (Newman-Keuls multiple comparison test). Cells treated or not with 100 nM of LTB_4_ were infected with *A. xylosoxidans* (MOI 30:1), incubated for 30, 120 or 360 minutes and submitted to quantification of *Defa1* mRNA expression by Real-Time PCR (**c**). One representative experiment out of two is shown as mean ± S.E.M. (n = 6–8 wells), ^#^ *p* < 0.05 for AMJ2-C11 infected *versus* LTB_4_ treated and infected AMJ2-C11 cells using Two-way analysis of variance (Bonferroni post-test).

To assess the mechanism by which LTB_4_ confers resistance to infection with *A. xylosoxidans*, we first evaluated the bacterial phagocytosis. For this purpose, AMJ2-C11 cells (murine alveolar macrophage cell lineage) were pre-treated or not with increasing concentrations of LTB_4_, followed by infection with FITC-labelled *A. xylosoxidans*. We observed that LTB_4_ did not affect the percentage of FITC^+^ cells (Fig. 7a) or MFI of infected AMJ2-C11 (data not shown), indicating that LTB_4_ did not increase phagocytosis of FITC-labelled bacteria. Next, we evaluated the ability of AMJ2-C11 to kill *A. xylosoxidans* in the presence or absence of exogenous LTB_4_. We observed that at 6 h after incubation, AMJ2-C11 cells pre-treated with 100 nM of LTB_4_ enhanced the bactericidal activity against *A. xylosoxidans* (Fig. 7b). At 24 h after incubation, all concentrations of LTB_4_ enhanced killing of *A. xylosoxidans* by AMJ2-C11 cells (Fig. 7b). Once LTB_4_ is known to induce pathogen killing by increasing the production of AMPs^18,24^, we determined the expression of *Defa1* in infected AMJ2-C11 cells. Notably, we detected that pre-treatment of AMJ2-C11 cells with LTB_4_ increased the expression of *Defa1* at 2 and 6 h post-infection with *A. xylosoxidans* (Fig. 7c).

### LTB_4_ potentiates the gene and protein expression of α-defensin-1 in the infected lungs

**Figure 8:**
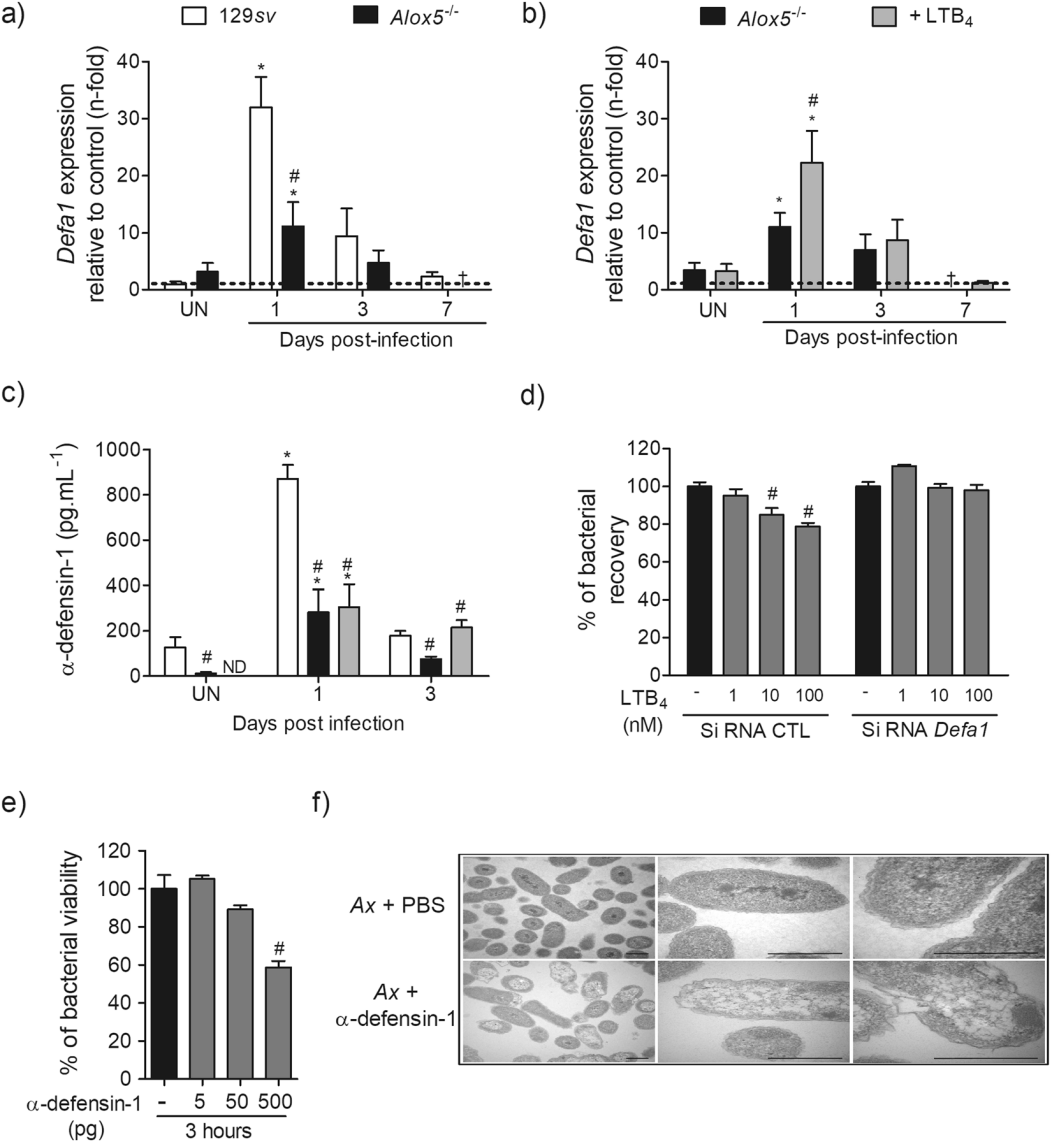
LTB_4_ induces and potentiates the release of the antimicrobial peptide alpha-defensin-1. qRT-PCR was used to evaluate Defa1 mRNA expression in lung parenchyma of wt, *Alox5* ^−/−^ and LTB_4_ treated *Alox5* ^−/−^ mice at 1, 3 and 7 days post-infection (**a**,**b**) and for quantification of α-defensin-1 peptide by ELISA in BALF (**c**). Data are mean ± S.E.M of three independent experiments (n = 4–15 mice per group at each time-point) for qRT-PCR and of one independent experiment (n = 4 mice per group at each time-point) for ELISA assay. *p < 0.05 for untreated (UN) *versus* infected mice, ^#^ *p* < 0.05 for 129*sv versus Alox5* ^−/−^ or *Alox5* ^−/−^ *versus Alox5* ^−/−^ + LTB_4_ using one-way analysis of variance (Newman-Keuls multiple comparison test). AMJ2-C11 cells were submitted to silencing of α-defensin-1 mRNA using siRNA. After 36 h of transfection, cells were pre-treated or not with LTB_4_ and then infected with *A. xylosoxidans* (MOI 5:1), and submitted for killing evaluation at 6 h (**d**). Data are mean ± S.E.M of one independent experiment (n = 5), ^#^ *p* < 0.05 for AMJ2-C11 infected *versus* LTB_4_ treated and infected AMJ2-C11 cells, using one-way analysis of variance (Newman-Keuls multiple comparison test). 10^5^ CFU of *A. xylosoxidans* incubated for 3 h, with crescent concentrations (5, 50 and 500 pg) of recombinant α-defensin-1 peptide were submitted to viability assay by resazurin metabolism (**e**). Data are mean ± S.E.M of one independent experiment (n = 5), ^#^ *p* < 0.05 for *Ax versus* α-defensin-1 treated *Ax* cells using one-way analysis of variance (Newman-Keuls multiple comparison test). Transmission electron microscopy of *A. xylosoxidans* (10^9^ CFU/ml) incubated with 500 pg of recombinant α-defensin-1 peptide for 2 h (**f**). *Bars* represent 1 µM.

Next, we determined the gene expression and secretion of α-defensin-1 in the lungs of infected 129*sv* and *Alox5* ^−/−^ mice. Compared to uninfected animals, pulmonary infection with *A. xylosoxidans* significantly increased *Defa1* expression in both strains of animals. However, 5-LO deficiency compromised the expression of *Defa1* induced by infection (Fig. 8a). Next, we assessed if treatment with LTB_4_ could restore the expression of *Defa1* in lungs of *Alox5* ^−/−^ mice. We observed that at the 1^st^ day post-infection, treatment with LTB_4_ rescued the expression of *Defa1* in lungs of infected *Alox5* ^−/−^ mice (Fig. 8b). We also evaluated the secretion of α-defensin-1 in BALF of both strains of animals. We observed that uninfected *Alox5* ^−/−^ mice present less α-defensin-1 when compared with uninfected 129*sv* mice (Fig. 8c). When infected with *A. xylosoxidans*, 129*sv* produce increased levels of α-defensin-1 compared to infected *Alox5* ^−/−^ mice, on 1^st^ day post-infection (Fig. 8c). At the 3^rd^ day post-infection, *Alox5* ^−/−^ mice treated with LTB_4_ exhibited similar amounts of α-defensin-1 compared to untreated 129*sv*-infected mice.

### The clearance of *A. xylosoxidans* induced by LTB_4_ depends on α-defensin-1 activity

To prove that increased killing of *A. xylosoxidans* induced by LTB_4_ is dependent on α-defensin-1 activity, we first silenced α-defensin-1 mRNA in AMJ2-C11 cells, and pre-treated the silenced cells with LTB_4_ before the infection with *A. xylosoxidans*. We observed that silenced AMJ2-C11 cells were unable to kill *A. xylosoxidans*, even in presence of exogenous LTB_4_ (Fig. 8d). Following, we incubated 1 × 10^5^ bacilli of *A. xylosoxidans* with increasing concentrations of recombinant α-defensin-1, and after 3 h we evaluated the antimicrobial activity of α-defensin-1. We observed a reduction of 40% in bacteria viability incubated with higher concentrations (500 pg) of recombinant α-defensin-1 (Fig. 8e). To determine how α-defensin-1 affects the viability of *A. xylosoxidans*, we performed a transmission electron microscopy of the bacteria incubated with α-defensin-1. We observed that this peptide disrupts *A. xylosoxidans* cell wall (Fig. 8f).

## Discussion

*A. xylosoxidans*, a pathogen frequently recovered from the lungs of CF patients, is associated with impairment of lung function^4^. Here, we aimed to determine the role of LTB_4_ in *A. xylosoxidans* lung infection to identify better therapeutic strategies. First, we established a sublethal inoculum of *A. xylosoxidans* for i.t. infection of 129*sv* (wild-type) mice. We found that the sublethal dose of 2 × 10^8^ bacteria induced a strong, but self-limiting infection restricted to the lungs, which was cleared by the 7^th^ day post-infection. At this time point, we observed an almost complete bacterial elimination, indicating that immune competent hosts were able to control the lung infection. In different time-points, the infection of 129*sv* mice was characterized by significant neutrophil recruitment, oedema formation, and the production of TNF-α, MIP-1α, IL-1α, MCP-1, LTB_4_, PGE_2_, and α-defensin-1 in the lungs_._ Some of these results are in accordance with previously published reports demonstrating that chronic infection with *A. xylosoxidans* in humans was associated with increased production of TNF-α and IL-6^6,7^, and that CF patients presented higher concentration of LTB_4_ and PGE_2_ in body fluids such as airway secretions and sputum^6,8^.

Products of 5-LO activity are essential to mount an efficient immune response in several infectious and inflammatory conditions^14,15,17,19,25^. Therefore, we investigated the role of LTB_4_ during infection with *A. xylosoxidans*. Using *Alox5* ^−/−^ mice, we found that downstream mediators of 5-LO activity were crucial for survival, for the efficient clearance of *A. xylosoxidans*, and control of lung oedema. Indeed, 5-LO deficiency results in similar phenotypes during several infectious or inflammatory diseases^13,15,17,19,26,27^. To confirm the essential and protective role of LTB_4_ in lung infection with *A. xylosoxidans*, we used two strategies. First, a pharmacological restoration of LTB_4_ function in the susceptible *Alox5* ^−/−^ infected mice, which almost abolished mortality induced by infection, promoted efficient clearance of *A. xylosoxidans* from lungs, and reduced lung oedema. Second, we performed a pharmacological inhibition of LTB_4_ function in the immune competent 129*sv* mice by the administration of U-75302, a potent and specific BLT_1_ receptor antagonist^19^. Inhibition of LTB_4_ signalling in *A. xylosoxidans*-infected 129*sv* mice significantly dampened the host response against the bacteria, increased mice mortality induced by infection, augmented bacterial load and lung oedema. These data agree with previous reports from our laboratory, demonstrating that the administration of LTB_4_-loaded microspheres enhances the lung immune response against *Histoplasma capsulatum* ^28^. In addition, i.n. administration of soluble LTB_4_ decreases lung oedema and abrogates mortality induced by scorpion venom^19^. Several groups, including ours, have postulated that the balance between LTB_4_ and PGE_2_ is relevant to the control of infectious and inflammatory diseases^19,29–31^. However, the same process is not apparent during infection with *A. xylosoxidans*. Indeed, we inhibited PGs production and Cysteinyl leukotrienes (CysLTs) signalling by treating infected mice with indomethacin or montelukast respectively (data not shown). We did not observe differences in the survival of treated mice, suggesting that during infections with *A. xylosoxidans*, PGs and CysLTs did not contribute to changes in bacterial burden, oedema formation, or to the control of leukocyte effector functions, as reported for other conditions^19,32,33^. The irrelevance of PGs is also supported in part by our finding that infection with *A. xylosoxidans* induced PGE_2_ only in the late stages of infection in 129*sv* mice, coinciding with mononuclear cell infiltration, a relevant source of this lipid mediator^34,35^. Overall, these data indicate that LTB_4_ is the predominant 5-LO product involved in the control of infection by *A. xylosoxidans*, and suggest that neutrophils and resident macrophages are the major source of this lipid mediator^35,36^.

The LTB_4_/BLT_1_ axis is essential for optimal phagocytosis and killing of *H. capsulatum* ^37^ and *S. pyogenes* by macrophages^15^. In contrast, LTB_4_ does not seem to increase the phagocytosis of *A. xylosoxidans* by alveolar macrophages, since the treatment of AMJ2-C11 cells with exogenous LTB_4_ did not increase the uptake of FITC-labelled bacteria (Fig. 7a). These contrasting results might result from differences in the receptors involved in the bacterial engulfment by the cells, as previous studies demonstrated a crucial role of LTB_4_ in FcRγ-mediated phagocytosis^37–39^. Although LTB_4_ did not affect phagocytosis of non-opsonized *A. xylosoxidans* by AMJ2-C11, exogenous LTB_4_ was crucial for clearance of *A. xylosoxidans* infection at 6 or 24 h post-infection *in vitro*. These results support our *in vivo* data and other works showing that LTB_4_ potentiates macrophage microbicide function^15,26,40^. Some of these studies associate the LTB_4_-depedent microbicide capacity with the generation of nitric oxide^40^, reactive oxygen species^15,26,40^ or release of AMP^18,41^. Defensins compose an important family of AMPs, subdivided into two main subfamilies, the α- and the β-defensins produced by polymorphonuclear cells and epithelial cells^42–45^. Their synthesis and release are up-regulated by lipopolysaccharide, cytokines, growth factors, among other stimuli^44^. Alveolar macrophages from rabbits produce α-defensin^42^, thus we investigated whether the increased microbicide activity of AMJ2-C11 cells conferred by LTB_4_ was due the secretion of α-defensin-1. In fact, we found that LTB_4_ increased transcription of *Defa1* in infected AMJ2-C11 cells; furthermore, expression of *Defa1* was 65% higher in lungs of 129*sv* mice compared to *Alox5* ^−/−^ mice. Of interest, LTB_4_ administration to *Alox5* ^−/−^ mice restored the expression of *Defa1*, and at the 3^rd^ day post-infection, BALF of infected and LTB_4_ treated *Alox5* ^−/−^ mice exhibits comparable levels of α-defensin-1 to that of 129*sv* mice. To confirm whether the production of α-defensin-1 is the mechanism by which LTB_4_ improved the killing capacity of AMJ2-C11 cells, we silenced AMJ2-C11 cells using siRNA against *Defa1* and evaluated the killing capacity of LTB_4_-treated cells. The silencing of α-defensin-1 mRNA completely impaired the killing of *A. xylosoxidans* induced by LTB_4_. Thus, we conclude that the microbicide effect observed in LTB_4_-treated AMJ2-C11 cells was due enhanced production of α-defensin-1 conferred by LTB_4_ signalling. Finally, we proved that α-defensin-1 is cytotoxic to *A. xylosoxidans*. Using a low dose of α-defensin-1 we observed significant loss of bacterial wall integrity. The role of α-defensin-1 during viral and bacterial infections was described before^18,41^. However, ours results are the first to demonstrate that LTB_4_ increases murine alveolar macrophage effector functions by releasing α-defensin-1. Of note, divalent cations, plasma proteins and salts impair the interaction of defensins with the microbial cell wall, and inhibit cell wall permeabilization^44,46^. This is of great significance since in CF patients, as the CFTR mutation results in chloride imbalance and dysregulation of ion channels, including impaired sodium transport to extracellular space. These alterations may impair the host defence in CF patients due to inhibition of some defensins activity^47^. Despite of this fact, we observed that even in non-physiological salt concentration, α-defensin-1 retains its function against *A. xylosoxidans* (Supplementary Fig. S1a). Our results are first evidence of the role of LTB_4_ signalling in α-defensin-1 release during an opportunistic bacterial lung infection. In *Alox5* ^−/−^ mice, *A. xylosoxidans* may induce mortality due to deficiency and/or impaired activity of α-defensin-1.

In summary, our results demonstrate for the first time the crucial and multifaceted role of LTB_4_ during infection with *A. xylosoxidans*. This lipid mediator participates in innate immune response by controlling the α-defensin-1 production and consequent clearance of *A. xylosoxidans*; and by reducing lung oedema formation. These findings suggest that LTB_4_ may serve as a potential therapeutic agent against *A. xylosoxidans* infection, especially in CF patients.

## Methods

### Mice

Age-matched, (24–26 g) 12–15-wk-old male 5-LO-deficient (*Alox5* ^−/−^) and wild-type mouse strains, both from 129*sv* genetic background, were obtained from the Jackson Laboratory (Bar Harbor, ME, USA) and raised at the Faculdade de Ciências Farmacêuticas de Ribeirão Preto, Universidade de São Paulo (FCFRP/USP). Experiments were approved and conducted in accordance with the guidelines of Universidade de São Paulo for the Use and Care of Animals (Number 14.1.390.53.0).

### *A. xylosoxidans* strain

*A. xylosoxidans* strain LMG 1863 from the Belgian Co-ordinated Collections of Micro-organisms BCCM/LMG were grown on Brain Heart Infusion (BHI) broth agar (Difco, Detroit, MI, USA) at 37 °C for 18 h. The colonies were resuspended in sterile PBS and the number of bacteria was determined spectrophotometrically (optical density at 600 nm)^15^.

### Determination of sublethal inoculum of *A. xylosoxidans*

The 129*sv* mice were anesthetized by intraperitoneal (i.p.) administration of ketamine and xylazine (75 and 10 mg/kg of body weight, respectively) and infected intratracheally (i.t.) with 100 µL of increasing inoculums of *A. xylosoxidans* (2 × 10^8^; 4 × 10^8^; 8 × 10^8^ and 16 × 10^8^). Mortality rate and sublethal inoculum were estimated using the moving average interpolation method^20,21^. Infected mice were monitored daily and mortality was recorded during the next 14 days. The median lethal and sublethal doses were estimated as 6.2 × 10^8^ and 2 × 10^8^ bacilli, respectively (Supplementary Table 1).

### Intratracheal infection and treatments

Both wt and *Alox5* ^−/−^ mice were anesthetized and i.t. infected with sublethal inoculums of *A. xylosoxidans* as described above. When necessary, *Alox5* ^−/−^ mice were treated with LTB_4_ (Cayman, Ann Arbor, MI, USA) (50 ng/animal in 20 µL in PBS, thrice daily at 8 h intervals) and 129*sv* mice were intranasally (i.n.) treated with U-75302 (a BLT_1_ antagonist; Cayman, Ann Arbor, MI, USA) (50 ng/animal in 20 µL in PBS, thrice daily at 8 h intervals). The treatments started one day before infection with *A. xylosoxidans*. Vehicle-infected or treated (i.t and/or i.n.) mice were used as negative controls.

### Experimental design and analyses

In specific experiments, infected 129*sv* and *Alox5* ^−/−^ mice, treated or not, were monitored for 14 days to mortality rate determination. For kinetic experiments, anesthetized mice were euthanized by cervical dislocation exsanguinated by cardiac puncture and the bronchoalveolar lavage fluid (BALF) was collected at 1, 3 and 7 days post-infection for total and differential leukocytes counts^25^ and total protein measurements in the BALF supernatant (Coomassie reagent, Pierce Chemical, Rockford, IL, USA). In another set of experiments, lungs from all groups were collected at the above-mentioned time points without performing a BAL. After weighting and fractionating the lungs, the left upper lobe was processed for bacterial burden^15^ plating four serial 0.05 mL dilutions on BHI broth agar and incubating at 37 °C for 36 h. Results were expressed as colony forming units (CFU) per gram of lung. The right middle lobe was used for histological analyses following haematoxylin and eosin (HE) staining. ImageJ software (U.S. NIH, Bethesda, MD, USA) was used to calculate the lung area covered by infiltrating cells in five random photomicrograph sections (100x resolution), and tissue damage was calculated as described^48^. The right upper lobe was homogenized, and the supernatant used (Mixer Homogenizer IKA Labortechnik, Staufen, Germany)^17^ for TNF-α, IL-1α, monocyte chemoattractant protein-1 (MCP-1) and macrophage inflammatory protein-1α (MIP-1α) quantification by ELISA (R&D Systems, Minneapolis, MN, USA). The right lower lobe was homogenized in 1 mL of ultra-pure water (v/v), followed by the addition of 1 mL of methanol and the supernatant was diluted in 9 mL of ultra-pure water prior to purification using Sep-Pak C18 column (Waters, Milford, MA, USA). The eluted fraction was used for LTB_4_ and PGE_2_ quantification using EIA (Enzo Life Science, Farmingdale, NY, USA)^19^.

### Cell culture

Alveolar macrophages, AMJ2-C11 cell lineage was acquired from the Rio de Janeiro Cell Bank, Rio de Janeiro Federal University (BCRJ code 0039). The cells were maintained in DMEM supplemented with 10% SBF and 1% gentamicin and grown at 37 °C, 5% CO_2_. Prior to experiments the cells were removed using cell scrapers and the total numbers of cells were determined in a Neubauer chamber in trypan-blue 0.4% (Sigma-Aldrich, St. Louis, MO, USA).

### Phagocytosis, bacterial killing and gene expression assay

To perform phagocytosis assay, 2 × 10^8^ *A. xylosoxidans* were labelled with Fluorescein isothiocyanate (FITC) (Amresco, Solon, OH, USA) at 0.1 mg/ml and washed twice with sterile PBS 1x ^17,39^. 1 × 10^6^ AMJ2-C11 cells were plated on 24 wells culture plates, pre-treated for 15 minutes with LTB_4_ (1, 10 or 100 nM), infected with FITC-labelled *A. xylosoxidans* (multiplicity of infection - MOI 50:1 bacilli per macrophage) and incubated at 37 °C, 5% of CO_2_ for 2 h. After this period, the cells were transferred for a cytometry tubes on ice and washed with cold and sterile PBS 1x. At the time of data acquisition, the cells were mixed with 0.9 mL of trypan blue (4 mg/mL, sodium citrate 14.7 mg/mL, pH 4.4) to chelate fluorescence of non-ingested bacteria. Samples were analysed using a FACSCanto Flow Cytometer (BD Bioscience, San Jose, CA, USA), and the percentage and MFI of FITC^+^ cells were estimated using the FACSDiva software (BD Bioscience, San Jose, CA, USA). To perform killing assay 2 × 10^5^ AMJ2-C11 cells were plated on 96 wells culture plates, pre-treated for 15 minutes with LTB_4_ (1, 10 or 100 nM), infected with *A. xylosoxidans* (MOI 5:1 bacilli per macrophage) and incubated at 37 °C, 5% of CO_2_ for 6 or 24 h. Then, the cells were lysed with saponin (0.05%) and mixed with 10 μL of a resazurin solution (0.5 mg/mL) (Sigma-Aldrich, St. Louis, MO, USA). The plate was incubated for 8 h at 37 °C, 5% of CO_2_. The relative fluorescence unit (RFU) was measured by a fluorimeter microplate reader (SpectraMax Paradigm, Molecular Devices, Sunnyvale, CA) at 560–590 nm. The RFU of untreated and infected cells were considered 100% bacterial recovery and the other groups were calculated relative to this group^49^. For gene expression assays, 5 × 10^5^ cells were plated on 24 wells culture plates, pre-treated for 15 minutes with LTB_4_ (1, 10 or 100 nm), infected with *A. xylosoxidans* (MOI 30:1 bacilli per macrophage) and incubated at 37 °C, 5% of CO_2_ for 30, 120 and 360 minutes. After this period the plate was centrifuged, the supernatant was removed, and the cells stored at −80 °C until RNA isolation.

### Quantitative polymerase chain reaction with reverse transcription

Total RNA was extracted according to manufacturer’s recommendations (Purelink, Ambion Invitrogen, Carlsbad, CA, USA), quantified by fluorometric method (Qbit, Invitrogen, Carlsbad, CA, USA) and the complimentary DNA (cDNA) was synthesized from 1 μg of total RNA (High Quality cDNA Reverse Transcriptase Kit, Applied Biosystems, Carlsbad, CA, USA). Fifty nanograms (*in vivo*) or a hundred nanograms (*in vitro*) of total cDNA was amplified by quantitative reverse transcriptase-polymerase chain reaction (qRT–PCR) using TaqMan primers for *Defa1* (Mm02524428_g1), in a StepOne Plus machine (Applied Biosystems, Foster City, CA, USA). *Gapdh* (Mm99999915_g1) and *Actb* (Mm00607939_s1) were used as reference genes (TaqMan^®^ Gene Expression Assay, Applied Biosystems, Carlsbad, CA, USA). Reaction conditions were applied as previously described^19^. The gene expression was normalized to the expression levels of *Actb* and *Gapdh* and the ΔΔCt method was used for the data analysis. The expression data was presented as *n-fold* difference relative to the control group and their average values were set as 1^19,49^.

### RNA interference using silencing RNA (siRNA)

1 × 10^4^ AMJ2-C11 were plated on 96 wells culture plate and maintained at 37 °C, 5% CO_2_ in serum and antibiotic free DMEM medium until 80% confluence (18–24 h). The cells were transfected with 40 pmol of *α-defensin siRNA (m)* (sc-40476) or *control siRNA-A* (sc-37007) using transfection reagent and Opti-MEM reduced serum medium (Santa Cruz biotechnology, Santa Cruz, CA, USA). After 36 h of transfection, cells were pre-treated or not with LTB_4_ (1, 10 or 100 nM; Cayman, Ann Arbor, MI, USA) for 15 minutes and then infected with *A. xylosoxidans* (MOI 5:1) for evaluation of killing for 6 h, as described above on killing assay. Efficiency of transfection was 85% for α-defensin, as determined by qRT–PCR. Controls included non-targeting siRNA (scrambled and fluorescent) and no siRNA (with and without lipofectamine).

### ELISA for mouse α-defensin-1 detection

The supernatant of BALF from 129*sv*, *Alox5* ^−/−^ and *Alox5* ^−/−^ treated with LTB_4_, uninfected and infected with *A. xylosoxidans* at 1 and 3 days post-infection were stored at −80 °C until performed the assay. At the day of quantification, 100 µl of BALF were added in respectively well and the assay was performed according to the manufacturer’s recommendations (SEB705Mu – Cloud-Clone Corp. Houston, TX, USA).

### Antimicrobial assay

10^5^ CFU of *A. xylosoxidans* were incubated with crescent concentrations (5, 50 and 500 pg) of recombinant α-defensin-1 peptide standard from ELISA kit (SEB705Mu – Cloud-Clone Corp. Houston, TX, USA), in PBS 1x supplemented or not with NaCl (150 mM) containing 1% (v/v) BHI (Difco, Detroit, MI, USA) in 96 well plate for 3 h at 37 °C. Then, we added 10 μL of a resazurin solution (0.5 mg/mL) (Sigma, St. Louis, MO, USA) (_VF_ = 200 µl) and we incubated the plate at 37 °C for 8 h. The RFU was measured by fluorimeter microplate reader (560–590 nm) (SpectraMax Paradigm, Molecular Devices, Sunnyvale, CA, USA)^49^.

### Transmission electron microscopy of bacteria

10^9^ CFU of *A. xylosoxidans* were treated with 500 pg of recombinant α-defensin-1 peptide standard from ELISA kit (SEB705Mu—Cloud-Clone Corp. Houston, TX, USA), in 0.5 ml of PBS 1x containing 1% (v/v) BHI for 2 h at 37 °C. Next steps were performed as previously described^50^. Bacteria were examined with JEM-100CX II electron microscope (JEOL, Peabody, MA, USA).

### Statistical analyses

Data were tested for Gaussian distribution using the D’Agostino-Pearson normality test. Mean values were compared between groups by a one-way or two-way analysis of variance followed by Newman-Keuls or Bonferroni multiple comparison tests. In some cases, student’s *t*-test was used. Survival differences between groups were calculated using a log-rank test. Analyses were performed using the Prism 6 software (GraphPad Prism, La Jolla, CA, USA). Significances are indicated for *p* < 0.05.

## Electronic supplementary material

Supplementary Information
