# Supplementary material for: Leukotriene B4 is essential for lung host defence and alpha-defensin-1 production during Achromobacter xylosoxidans infection
Source: Sci Rep. 2017 Dec 15;7:17658. doi: 10.1038/s41598-017-17993-9 (PMC5732241; doi:10.1038/s41598-017-17993-9)
Supplement: Supplementary file 1 — Supplementary Information [file 41598_2017_17993_MOESM1_ESM.pdf]

Leukotriene B_4 is essential for lung host defence and alpha-defensin-1 production during *Achromobacter xylosoxidans* infection

Morgana K. B. Prado[1], Gisele A. Locachevic[1], Karina F. Zoccal[1], Francisco W. G. Paula-Silva[1], Caroline Fontanari[1], Joseane C. Ferreira[1], Priscilla A. T. Pereira[1], Luiz G. Gardinassi[1], Simone G. Ramos[2], Carlos A. Sorgi[1], Ana Lúcia C. Darini[1] and Lúcia H. Faccioli[1*].

[1]Departamento de Análises Clínicas, Toxicológicas e Bromatológicas, Faculdade de Ciências Farmacêuticas de Ribeirão Preto, Universidade de São Paulo, Ribeirão Preto, São Paulo, Brazil. [2]Departamento de Patologia e Medicina Legal, Faculdade de Medicina de Ribeirão Preto, Universidade de São Paulo, Ribeirão Preto, SP, Brazil. *Correspondence and requests for materials should be addressed to L.H.F. (email: faccioli@fcfrp.usp.br)

Supplementary data

Supplementary table 1: Parameters used for the determination of lethal and sublethal inoculla by the moving average interpolation method

CFU	Total animals	Number of deaths
2×10^8	6	0
4×10^8	6	1
8×10^8	6	4
16×10^8	6	6

CFU: colony forming unit.

Figure S1A

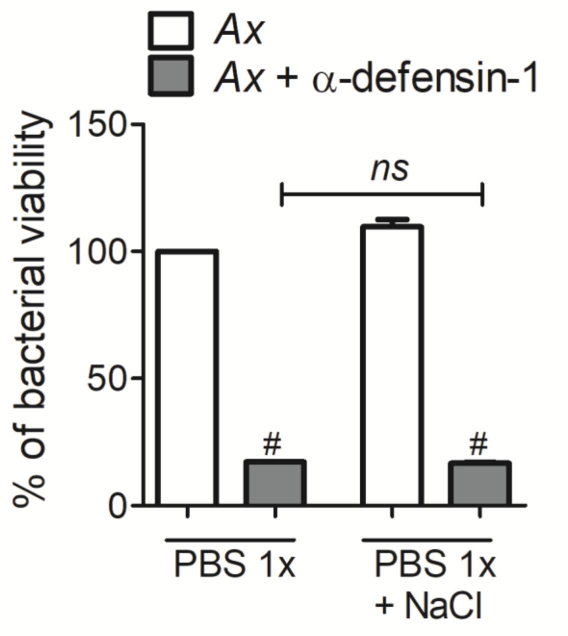

Figure S1A: 10^5 CFU of *A. xylosoxidans* were incubated for 3 hours with 500 pg of recombinant α-defensin-1 peptide in the presence or absence of 150 mM of NaCl and then submitted to viability assay by resazurin metabolization. Data are mean ± S.E.M of one independent experiment (n = 3), #: $p < 0.05$ for *Ax* versus α-defensin-1 treated *Ax* cells using one-way analysis of variance (Newman-Keuls multiple comparison test).
